# Experiences developing structural biology tools with AI coding assistance

**DOI:** 10.1107/S2059798326010211

**Published:** 2026-10-19

**Authors:** Oleg V. Sobolev, Dorothee Liebschner, Paul D. Adams

**Affiliations:** a Molecular Biophysics and Integrated Bioimaging Division Lawrence Berkeley National Laboratory Berkeley California USA; b Department of Bioengineering University of California Berkeley Berkeley California USA; European Bioinformatics Institute, United Kingdom

**Keywords:** AI-assisted software development, crystallographic information file, *cctbx*, methods development, PDBx/mmCIF parsing, structural biology software validation.

## Abstract

Large language models (LLMs) offer significant potential to accelerate scientific software development, yet their application to structural biology algorithms requires rigorous evaluation. This challenge is examined through the development of *xcif*, a new native parser for the crystallographic information file (CIF) format, designed to replace a stable but slow parser (*ucif*) in the Computational Crystallography Toolbox (*cctbx*). Using an expert-led, artificial intelligence (AI)-assisted workflow, implementation time was reduced from an estimated several months to approximately one week of active human effort. However, this efficiency relied entirely on domain knowledge to specify constraints, intercept plausible but flawed machine-generated code and design adversarial tests. The machine assistance did not replace programming expertise but rather shifted the human engineering burden from writing code to independent validation. Applying the new *xcif* parser across a corpus of approximately 550 000 archive files confirmed that it produces output identical to both the legacy implementation and *GEMMI*, a modern high-performance parser. *xcif* parses roughly an order of magnitude faster than the legacy *cctbx* parser and 1.3–1.5 times faster than *GEMMI*, while reducing peak memory use for larger files. This case study demonstrates that AI assistance provides a highly practical, cost-effective pathway for the renewal of mature structural biology infrastructure, provided that the development is strictly governed by expert oversight and objective verification.

## Abstract 2

Artificial intelligence (AI)-assisted development can significantly reduce the effort of coding scientific tools and software. At the same time, the approach requires detailed oversight, as well as domain knowledge and codebase expertise. The conditions under which AI assistance is reliable, the kinds of mistakes coding LLMs can make and why expert judgment is necessary are examined through the example of developing a new CIF file parser that focuses particularly on speed of execution.

## 1. Introduction

Developing and maintaining the algorithms and infrastructure that enable computational structural biology is a highly labour-intensive process. Scientific software carries stringent correctness requirements; a hidden flaw in a core algorithm or data parser can silently corrupt a result that is later published. Recently, large language models (LLMs) have emerged as interactive coding tools capable of producing working programs from natural-language directions (Chen *et al.*, 2021 ▸). For scientific computing, this presents an opportunity and a hazard in equal measure. The opportunity is that software can be produced more efficiently, bringing the renewal of essential tools within the budget of small academic teams. The hazard is that language models are prone to domain-specific errors (Chen *et al.*, 2021 ▸; Liu *et al.*, 2023 ▸). The practical question is therefore not whether these models can generate code, but how they can be safely applied to correctness-critical scientific software, what failure modes they introduce and what human expertise must still be supplied. These questions are best examined not in the abstract, but through a concrete case study where the criteria for correctness are unambiguous.

This article examines the artificial intelligence (AI)-assisted development of a foundational structural biology tool: a high-performance parser for the crystallographic information file (CIF; Hall *et al.*, 1991 ▸), its macromolecular extension PDBx/mmCIF (Bourne *et al.*, 1997 ▸; Westbrook *et al.*, 2022 ▸) and other derived variants and formats. These are the formats in which structural data are archived, and increasingly the formats read at every step of refinement, validation and rebuilding. Mandatory deposition in PDBx/mmCIF has been in effect since 2019 (Adams *et al.*, 2019 ▸), the legacy PDB format is being retired, and from July 2027^1^ new Protein Data Bank (Berman *et al.*, 2000 ▸) entries will be distributed only in PDBx/mmCIF and Protein Data Bank Markup Language (PDBML). Parsing these files sits on the critical path of data ingestion. The Computational Crystallography Toolbox (*cctbx*; Grosse-Kunstleve *et al.*, 2002 ▸) is the open-source library underlying *Phenix* (Liebschner *et al.*, 2019 ▸) and other crystallographic packages (Winter *et al.*, 2018 ▸; Dolomanov *et al.*, 2009 ▸; Winter, 2010 ▸). Its CIF parser, provided since 2011 through *iotbx.cif* (Gildea *et al.*, 2011 ▸) and here called *ucif*, has served the community reliably for more than a decade. Its one deficiency is speed: it is an order of magnitude slower than the fastest current parser, *GEMMI* (Wojdyr, 2022 ▸). As mmCIF becomes the format read at every step, *ucif*’s speed deficit becomes more obvious, making it a representative instance of a situation common across mature scientific software: a correct, stable, but slow component whose replacement is desirable yet has never been worth the engineering cost it would demand. Writing a CIF parser is an ideal test case for expert-led, AI-assisted development of critical software. Success is clearly defined because the new output can be checked for exact agreement against trusted, existing tools (*ucif* and *GEMMI*). Furthermore, a fixed, published specification firmly defines the rules, leaving little of the open-ended ambiguity that complicates the development of entirely novel methods.

Replacing such a component traditionally presents a difficult choice between adopting an external library or writing a native one. Adopting an external parser introduces long-term maintenance burdens and requires data conversion between the external parser’s representation and the native data structures of *cctbx* at every point of use (Section 3). Writing a native parser avoids that conversion entirely, but was estimated to be a multi-month undertaking whose deliverable is not itself a new scientific result. AI assistance alters this cost–benefit calculation by absorbing the bulk of the implementation labour, provided that the resulting code can be rigorously validated (Section 4).

This work reports two contributions relevant to structural biology software development. Firstly, it presents *xcif*, a native CIF and PDBx/mmCIF reader integrated into *cctbx* that replaces the existing *ucif* parser. It preserves API compatibility through a dedicated compatibility path while enabling a native path that hands data directly to *cctbx* without rebuilding the legacy data structures. Correctness is established by exact, value-level comparison against *ucif* and *GEMMI* across archive-scale validation sets, and performance is quantified in matched-output benchmarks. Secondly, the article documents a transferable, expert-led workflow for using AI assistance to develop correctness-critical scientific infrastructure. This workflow emphasizes adversarial planning, explicit interception points for human architectural judgment, test-driven implementation and independent cross-parser validation, demonstrating how developer effort can be reduced dramatically while domain judgment, codebase understanding, rigorous testing and accountability remain human responsibilities.

## 2. AI-assisted development workflow

The work of building the new CIF parser fell into two distinct phases: planning and implementation. The planning phase was where the developers (the authors of this work) decided what code should be built, where it should go and how it should fit into the existing codebase, and recorded those decisions as a specification for the LLM to follow. The implementation phase was the stage where the plan was translated into actual code changes. The developers’ expertise spans the CIF format and crystallography (the scientific domain), together with the *cctbx* codebase and software engineering (the practice domain), a combination characteristic of research software engineering. Each has more than a decade of experience developing structural biology software and contributing to *cctbx* and *Phenix*; the lead developer also holds an engineering degree in software systems development. This description characterizes the expertise available in the present case but should not be interpreted as a minimum experience threshold for applying the workflow.

### 2.1. Planning

The planning phase was carried out through the chat interfaces Claude Opus 4.6/4.8 and Gemini 3.1/3.5 Pro. It began with a specification, which inherently required domain expertise. The AI models do not know which constraints a crystallographic parser must honour, which optimizations are sound for this format and which are traps, or how the result must integrate with an existing library; the developers must supply all of this before any code is written. The planning was conducted adversarially (Madaan *et al.*, 2023 ▸; Shinn *et al.*, 2023 ▸). Designs drafted with one model (Claude Opus) were submitted to the other (Gemini Pro) in a separate session sharing no context with the first, under an explicit instruction to identify weaknesses rather than endorse the design (Du *et al.*, 2023 ▸); a representative instruction was of the form: ‘You are a senior engineer tasked with critically reviewing this plan. Your goal is to find every potential flaw, unclear assumption, or workflow violation. Be specific and constructive; do not give generic praise.’ Transfer between the two models was manual: review findings were carried back into the drafting session by the developer, who added his own concerns in the same step (*e.g.* ‘You seem to forget about a huge class of CIF files: structure factors’) and adjudicated each finding: accepted, rejected as inapplicable to the format, or deferred to experiment. The same adversarial stance was applied to generated code, for example, by opening a fresh session seeded with a stated suspicion (‘I suspect the implementation for memory-mapped file I/O is missing, incomplete, or not correctly wired…’) and instructing the model to audit rather than extend. The quoted instructions above are drawn from retained session logs; because not every session was archived, they illustrate the procedure rather than provide an exhaustive record.

Disagreements between the models arose mainly on domain-specific questions that neither model could resolve authoritatively and were resolved by the developer (Liu *et al.*, 2023 ▸). Model agreement was not sufficient evidence of correctness: in the loop-storage decision (Table 1 ▸) both models converged on a design that the developer overruled. The rejected optimizations were typically sound for general-purpose text processing but not for this format or codebase; some were identified during adversarial review, whereas others required a developer familiar with *cctbx*’s integration constraints. Table 1 ▸ collects such cases from across the work and identifies how each was detected.

**Figure 1:**
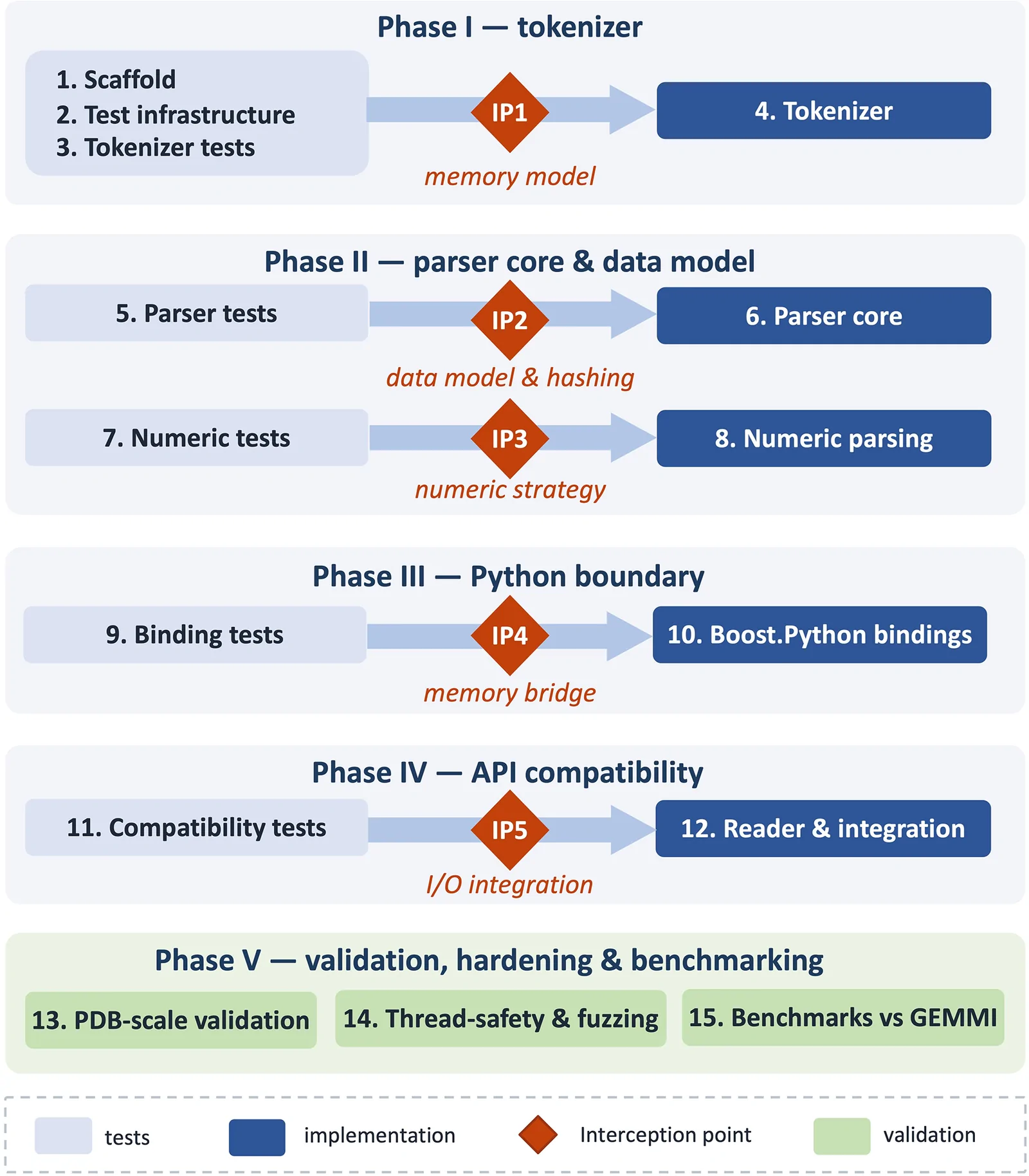
*xcif* development roadmap illustrating the five phases, the 15 steps and the five interception points.

The output of the planning phase was the implementation prompt reproduced in Supplementary Information S1: a single document fixing the hard constraints – the language standard the code was restricted to, the build machinery it had to integrate with and the existing interface it had to remain compatible with – together with a 15-step roadmap grouped into five phases of distinct concern. Crucially, it included five interception points at which execution was required to halt and present design options for a human decision rather than allowing the model to choose for itself (Fig. 1 ▸). These points fell before the most consequential architectural commitments (the tokenizer memory model, the data model and hashing strategy, numeric parsing, the Python–C++ memory bridge and I/O integration) and are documented with their outcomes in Supplementary Information S2. This prompt established the boundary within which models’ capabilities became useful; assembling it required knowing the problem and the codebase well enough to anticipate the AI models’ likely mistakes before they were made. It served as the comprehensive specification that the second phase executed against.

### 2.2. Implementation

Generating code through a structured, staged process has been reported to produce better results than generating it directly from a single prompt, particularly when complex tasks are decomposed into smaller units (Wei *et al.*, 2022 ▸; Zhou *et al.*, 2023 ▸; Almorsi *et al.*, 2025 ▸) and intermediate outputs are validated (Chen *et al.*, 2023 ▸). The implementation was carried out using an AI coding agent (Claude Code). By ‘agent’, we mean an assistant that works through multiple steps on its own (editing files, running builds and tests, and responding to the results) rather than simply generating code. This autonomy was deliberately limited. The agent could decide how to implement and test each roadmap step within the constraints of the specification, but it had to stop at predefined checkpoints where architectural decisions were left to the developers. It worked through the roadmap one step at a time, with no stage begun before the previous one was complete. This approach served the further purpose of creating defined points at which the developers reviewed intermediate output. At each of the five interception points the agent paused and presented its architectural options, and the developers chose; the choices made and the reasoning behind them are summarized in a decision record compiled at the end of implementation (Supplementary Information S2).

Within each step, the agent worked under a test-driven discipline (Beck, 2003 ▸) prescribed by the implementation prompt (Supplementary Information S1) and executed by Claude Code: tests were written first, confirmed to fail against the existing code, implementation was added and the same tests were confirmed to pass. All tests, like the implementation, were AI-generated: approximately 7000 lines of tests against 2500 lines of implementation; the developers reviewed the tests but wrote none by hand. The tests fall into three groups: unit tests of the C++ core, from the tokenizer to numeric parsing with standard-uncertainty extraction; tests of the Python binding layer, including memory lifetime and GIL release; and comparison tests that parse identical inputs from synthetic and real PDB files through both *xcif* and the existing *iotbx.cif* reader, supplemented by thread-safety tests and a fuzz target. Because agent-written tests can pass without exercising the intended behaviour, and an agent may also misreport test results (Liu *et al.*, 2023 ▸), the developers verified early in the implementation that the reported testing was real: for a few tests, chosen simply as ones whose code under test was easiest to break deliberately, they temporarily introduced errors and confirmed that the tests failed. These spot checks confirmed that the tests actually ran and did what the agent claimed; they were deliberately not extended into a systematic audit (Jia & Harman, 2011 ▸), because the correctness argument of this work rests not on the test suite but on the independent cross-parser validation of Section 4.

The same principle governed the final review. The pull request was assessed by a *cctbx* maintainer (B. K. Poon; see Acknowledgements) who, like the developers, used the agent as a critic rather than a generator of code, with the developers deciding which of its findings warranted action. AI thus appeared on both sides of the review, as author and as critic, with a human adjudicating in each case, an arrangement that works only because the adjudicator brings knowledge that neither model has. The independent confirmation that the parser does what the agent claimed it does was ultimately established not by any account the agent gave but by checking its output, file by file, against two parsers developed entirely separately from it; that validation is the subject of Section 4.

The active human effort across planning, execution and review (inclusive of the validation work) was approximately seven working days, compared with a pre-AI estimate of several months by comparison with the development of *ucif*, whose implementation spans roughly 100 active development days in the public *cctbx* commit history over 2010–2011. The archive-scale correctness check was itself largely automated (Section 4.2) and would have been equally necessary for a manually written replacement, so it does not account for the difference; the saving reflects the implementation labour absorbed by the agent. This compressed timeline was only feasible because the developers were already fluent in both the CIF format and the *cctbx* codebase. The compute cost of the AI assistance, all of it in the implementation phase, was on the order of one hundred US dollars.

## 3. Description of the new CIF parser

The CIF parser, named *xcif*, is written in C++ with a Python interface and is built around two primary design principles. Firstly, parsed values are stored as references into the source buffer rather than as copies: the input file is memory-mapped, and each value is a view into it, so parsing performs almost no allocation. Second, *xcif* lives within *cctbx*, and its binding layer emits *cctbx*’s native data types directly (*scitbx* flex arrays^2^ and iotbx.cif.model-compatible objects^3^) rather than a private representation that callers would have to translate. The first principle yields parse-time speed and memory efficiency competitive with *GEMMI*; the second lets *cctbx* consume *xcif*’s output with no data-structure conversion, whereas a foreign library such as *GEMMI* produces a parse tree that must first be translated into *cctbx* equivalents. The implementation comprises approximately 2500 lines of C++ and Python source code.

A CIF parser processes a file and organizes its individual data values, such as atomic coordinates or atom names, in the computer’s memory. General-purpose parsers such as *GEMMI* typically create a new, independent memory copy for every single value. While this is a safe and standard approach for a standalone library, it requires substantial memory allocation when parsing structural files with millions of values. Instead of copying data, *xcif* uses a ‘view’ approach: it simply records the starting position and length of each value directly within the original file. The practical benefit of this design becomes clear when the parser hands the data off to the rest of the *cctbx* software. Because *xcif* points directly to the source file, numerical values can be converted straight into the final mathematical arrays that *cctbx* uses (native path described below), entirely skipping the creation of intermediate text copies. Furthermore, highly repetitive text columns, such as element symbols, can be deduplicated efficiently. If *cctbx* had instead relied on wrapping an external parser, the data would have to be copied by the parser first and then processed again by *cctbx*. Therefore, the primary performance advantage of *xcif* lies not in reading the text faster, but in avoiding the creation of millions of unnecessary intermediate data copies.

The new parser integrates into the broader software environment through two distinct pathways that trade compatibility against speed. The compatibility path reproduces exactly the data structures of the old parser, so existing tools benefit from faster parsing with no other changes; the native path skips that reproduction and delivers data straight into *cctbx*’s numerical arrays, which is faster still but requires the calling code to be updated. The compatibility path is designed as a seamless drop-in replacement for the old parser: by adding a single keyword (engine="xcif") to the existing *cctbx* CIF reader (iotbx.cif.reader()), the new parser mimics the exact data format produced by the legacy tool. This allows existing structural biology tools to benefit from faster parsing immediately, without requiring any other code updates. The second option is a faster ‘native path’. Rather than translating the data to match the legacy format, this path delivers numerical data directly into the program’s arrays, realizing the full speed benefits described above. The performance of both paths is evaluated in Section 4, noting that while the compatibility path is currently in active use, transitioning all existing tools to the faster native path remains a work in progress. Finally, the parser supports all standard CIF specifications required for PDB data, omitting only specific extended CIF2 features (Bernstein *et al.*, 2016 ▸) that do not appear in structural biology archives.

## 4. Validation and performance of the new CIF parser

In production scientific software, a new tool intended to replace a long-established component carries two obligations: it must produce identical results to the legacy tool, and it must measurably improve the property that motivated the replacement (*e.g.* speed). To evaluate the new *xcif* parser, we validated its ability to correctly read the variety of available CIF files and produce the same results as the *ucif* parser. In addition, we analysed its parsing speed and memory usage and compared it against the current state-of-the-art reference.

### 4.1. CIF files used to validate parsing and test performance

A robust crystallographic parser must be capable of reading all available forms of CIF data, including coordinate models, diffraction data, restraints and chemical components. Accordingly, validation was performed against the full PDB archive, which contains 252 426 coordinate files (mmCIF) and 193 791 structure-factor files as of April 2026. These files were obtained from the PDB and parsed in their original compressed formats (.cif.gz and .ent.gz) without preprocessing. This corpus was supplemented with the complete GeoStd library (Moriarty *et al.*, 2026 ▸) and the wwPDB Chemical Component Dictionary (Westbrook *et al.*, 2015 ▸), the latter included both as the single consolidated file (components.cif, 479.5 MB) and as the individual files into which it is split for the *Phenix* distribution. GeoStd and the split dictionary together amount to 102 688 files. Three IUCr/wwPDB dictionary files were also included: cif_core.dic (the IUCr core CIF dictionary), cif_mm.dic (macromolecular/mmCIF dictionary) and pdbx_v50.dic (wwPDB PDBx/mmCIF dictionary, extending the cif_mm dictionary). For the performance benchmark (Section 4.3) the file set from Wojdyr’s published *GEMMI* benchmark^4^ was used, supplemented with two additional large data files chosen to probe the upper tail of file sizes (PDB entries 5rhy and 5rob).

### 4.2. Correctness

Correctness was verified through a three-way, item-by-item comparison among *ucif*, *xcif* and *GEMMI*. For each file all three parsers were run via Python, and the extracted data were standardized to record block names, data labels and values in their original source order. Two parsers agree on a file if their representations match exactly, and *xcif* passes a file only if it agrees with both *ucif* and *GEMMI*. The comparison is deliberately strict: all three parsers must extract every piece of information in the file, in the same order, with text formatting such as quotes and spacing handled correctly; any single mismatched data value, reordered loop or missing data section fails the file.

The full-archive validation was executed in parallel across 50 cores in batches of 100 files, completing in approximately three hours for the coordinate set and three hours for the structure-factor set. Files larger than 100 MB when compressed (44 files in the structure-factor set) were compared exclusively between *xcif* and *GEMMI*, as *ucif* encountered memory limitations.

Across all approximately 550 000 files described in Section 4.1, *xcif* produced output identical to both *ucif* and *GEMMI*. For the 44 oversized structure-factor files on which only the *xcif*-against-*GEMMI* comparison was feasible, the two parsers agreed exactly on every file. The Chemical Component Dictionary and the three dictionary files likewise produced fully consistent output across all three parsers.

### 4.3. Performance

Benchmarks were run on a MacBook Pro (Apple M2 Pro, 32 GB RAM, macOS Sequoia 15.6.1) using the *Phenix* 2.1-6048 distribution. This version contains *cctbx* v2026.5 (https://github.com/cctbx/cctbx_project/releases/tag/v2026.5) and includes the versions of the *ucif* and *xcif* parsers tested here, as well as *GEMMI* v0.7.3 (https://github.com/project-gemmi/gemmi/tree/v0.7.3) and Python 3.9; each timing is the average of ten repeats after two warm-up runs, and peak resident set size was recorded per process.

Meaningful performance benchmarks require parsers that produce equivalent outputs; otherwise, timing differences reflect the extent of data processing rather than pure parsing efficiency. The two reference parsers yield different final data structures: *ucif* builds a Python iotbx.cif.model, whereas *GEMMI* produces a native C++ structure. The performance of *xcif* is therefore evaluated through two pathways, one matched to each. The compatibility path, *xcif_compat*, builds the same iotbx.cif.model as *ucif* and isolates the difference in parsing efficiency; for the native comparison, *xcif_native* and *GEMMI* are each timed through construction of their respective native C++ data structures. Comparing across these output types (such as *xcif_native* against *ucif*) would primarily measure the computational cost of building the Python model, not the raw parsing speed.

**Figure 2:**
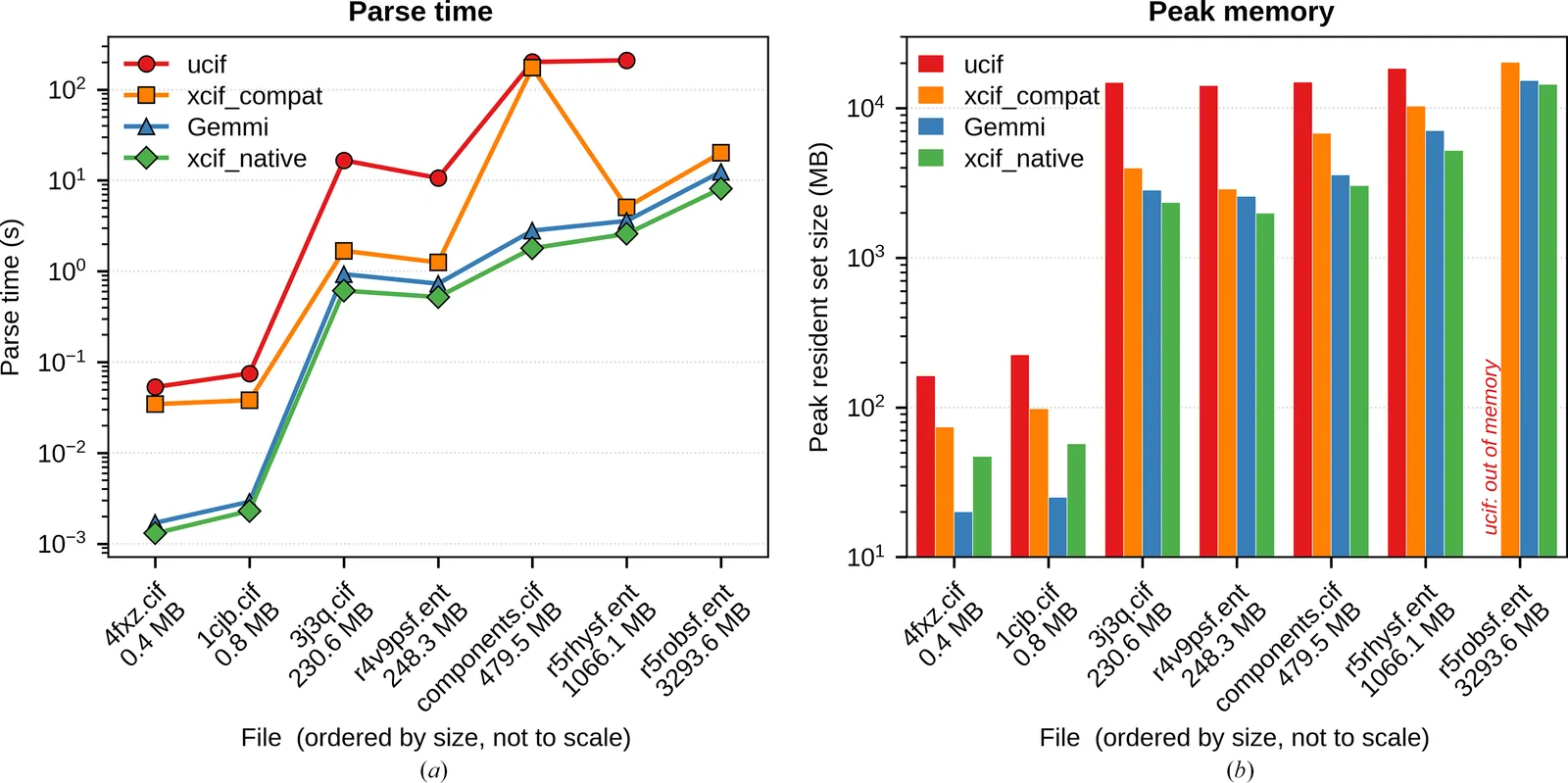
Parse time (*a*) and peak resident set size (*b*) for *ucif* (red), *xcif_compat* (orange), *GEMMI* (blue) and *xcif_native* (green) on CIF files of increasing size. Both vertical axes are logarithmic. The values are also listed in Supplementary Tables S1 and S2.

The parse time for all paths is shown in Fig. 2 ▸(*a*) and Supplementary Table S1. In the compatibility path, *xcif_compat* outperforms *ucif* by a factor ranging from roughly 1.5 on the smallest test file (4fxz.cif), where both are dominated by Python function-call overhead independent of file content, to 41.5 on a 1066 MB structure-factor file (PDB entry 5rhy). The Chemical Component Dictionary is an outlier in file structure, containing approximately 50 000 small data blocks, and here the per-block Python construction in iotbx.cif.model dominates the execution cost of both parsers. This bottleneck is a property of the Python data model, not of the parser, and it is exactly what the native path avoids.

In the native path, *xcif_native* is 1.3–1.5 times faster than *GEMMI* on every file in the benchmark that is large enough to exceed the measurement-noise floor, and this performance margin stays roughly constant across the tested size range. This characteristic stems from how data values are represented in memory: each value is a 16-byte view into the memory-mapped source file, avoiding per-value memory allocation during parsing. In contrast, *GEMMI* creates a new, independent copy in memory for every value it extracts. On files with millions of values, avoiding these continuous memory allocations yields the observed reduction in parsing time. This design choice similarly impacts peak memory usage: *xcif_native* uses 5–27% less peak memory than *GEMMI* on files at or above ∼200 MB (Fig. 2 ▸*b*, Supplementary Table S1). On the two smallest files its memory footprint exceeds *GEMMI* ’s by a fixed ∼30 MB offset; *xcif* is part of *cctbx*, so loads *cctbx*’s supporting libraries, which standalone *GEMMI* does not; this is a fixed environmental overhead, not a property of the parser itself. Separately, *ucif*’s peak memory is up to five times that of *xcif_compat* when producing identical Python data structures, reflecting runtime overhead rather than the data-structure size.

The practical impact of the native integration extends beyond raw parsing speed. Existing scripts and applications that parse data to iotbx.cif.model become substantially faster with a single keyword change (engine="xcif") for workloads where parsing is the primary bottleneck. Furthermore, workflows that can avoid the Python data model entirely execute one to two orders of magnitude faster, and processing files of arbitrary size becomes computationally feasible for routine workflows. For example, a 3.3 GB structure-factor file parses in 8.1 s through the native API. This native path is not yet integrated into all of *cctbx*’s production tools; the performance figures establish the potential of this representation, and realizing it across the software suite is planned as future work.

## 5. Discussion

The development of *xcif* demonstrates that while artificial intelligence can substantially accelerate the implementation of scientific software (Peng *et al.*, 2023 ▸), producing a trustworthy result relies heavily on human domain expertise. Throughout the planning, coding and review phases, the AI model served as a highly capable but fallible assistant. As detailed in Table 1 ▸, the model frequently proposed optimizations that are standard in general software engineering but inadequate for the specific requirements of the CIF parsing task, such as failing to account for standard-uncertainty notation or introducing operating-system locale dependence. Catching these domain-specific errors required a developer who already understood the CIF format and the *cctbx* codebase. As a consequence, the nature of the developer’s work fundamentally shifted. In traditional development, much of the time is typically spent writing and debugging code. In an artificial intelligence-assisted workflow, routine implementation effort is largely absorbed by the LLM, but the time spent on adversarial planning, designing validation tests and reviewing generated code rises significantly. When an AI model generates the code, the developer does not acquire the deep familiarity that naturally comes from writing it manually, which makes rigorous, independent testing strictly necessary.

The reallocation of developer effort indicates where this AI-assisted approach is most readily applicable. Effectively specifying constraints, evaluating AI proposals and verifying correctness require three conditions: a clear specification of software behaviour, a domain expert capable of recognizing unsound architectural choices and an independent reference implementation for validation. These conditions are met in the development of format parsers, writers, data converters and similar foundational infrastructure, where specifications are firmly fixed and legacy implementations already exist. While it is likely that the development of entirely novel scientific methods can also benefit from AI assistance, the process presents greater challenges. The primary obstacle in novel software development is assembling a trustworthy test corpus without a pre-existing reference for comparison, although generating such validation data remains a standard requirement for publishing any new scientific method.

Verification requirements vary across scientific software, so it is helpful to place this work within a broader classification of research software. Under the role-based scheme of Hasselbring *et al.* (2025 ▸), *xcif* is research infrastructure software (specifically, a library) rather than modelling, simulation and data-analytics software or technology research software. The level of risk varies both between and within these categories. It depends mainly on two questions: how serious would a silent or undetected error be, and is there an independent standard against which the software can be checked? Presentational and interactive tools, such as scientific visualizations and graphical or command-line interfaces, tend to be lower risk because their mistakes are usually visible and inexpensive to correct. Small exploratory scripts present a different verification problem. They often lack a clear reference result, but their iterative use permits informal cross-checks against alternative visualizations, tables, descriptive statistics and the researcher’s developing understanding of the data; these safeguards are weaker in early-stage work, when prior expectations are limited. AI assistance can therefore save labour in these areas with relatively light oversight. Infrastructure libraries such as *xcif* require much greater care because an error may silently affect every downstream result. At the same time, they can be unusually straightforward to validate: in this case, a fixed specification and independent reference implementations provide strong standards for comparison. Other types of scientific software can be just as consequential but much harder to check. Modelling and simulation software may silently compromise published results while offering no independent reference output. Validation must then rely on carefully designed test cases and physical reasoning: the same difficulty we identify for entirely novel methods. Control-and-monitoring software and other technology research software used in cyber-physical settings introduce additional real-time and safety risks, raising the standard of verification further. AI assistance may offer similar efficiency gains across these categories, but the oversight needed to use it safely increases with the consequences of an error and depends on whether independent verification is possible. *xcif* is a case in which the stakes are high but the means of checking correctness are unusually strong. When no such independent standard exists, achieving the same confidence becomes considerably more difficult.

Renewing slow but functional scientific infrastructure traditionally presents a difficult choice between adopting an external software dependency or bearing the high development cost of writing a native replacement. For *cctbx*, a native CIF parser integrates cleanly but has long been difficult to justify due to the extensive implementation labour required. By absorbing the bulk of that coding effort, artificial intelligence made the native option feasible for a small academic team. This approach makes infrastructure renewal practical for projects that possess the requisite domain expertise but lack the months of dedicated developer time typically needed to replace legacy software that already works. Currently, *xcif*is a working parser in production use; however, while its compatibility mode is active, integrating its higher performance native path into the rest of *cctbx* remains to be done. This future work stands to benefit from the same guided, artificial intelligence-assisted approach, demonstrating how these tools can sustainably modernize the computational foundations of structural biology.

An implementation cost of roughly $100 invites comparison with developer salaries, but this figure reflects only the marginal cost of a single task, not the cost of maintaining the expertise on which the workflow depends. The approach described here is safe because it is directed by developers who already understand both the CIF format and the *cctbx* codebase in depth. That understanding is not innate; it has traditionally been acquired by writing, debugging and maintaining the kind of foundational code that an AI agent now produces. If entry-level implementation work is the route by which developers became the experts capable of specifying constraints, recognizing unsound proposals and designing adversarial validation, then automating that work risks eroding the expertise of reviewers on whom the method depends. This is a long-term sustainability concern that short-term efficiency gains can obscure: a team can save months on one component while quietly reducing the opportunities through which its next generation of developers would have been trained. We therefore view preserving human expertise as an explicit responsibility for groups adopting this approach, rather than as an automatic outcome. In practice, this means involving less-experienced developers directly in the parts of the workflow in which domain judgment develops instead of confining these roles to a shrinking pool of senior staff. Long-term sustainability therefore still requires funded junior positions, mentoring and hands-on participation in software development. Used thoughtfully, AI assistance can support training rather than displace it; used carelessly, it trades lasting research software engineering capability for immediate speed.

## 6. Conclusion

AI-assisted development significantly reduces the labour of implementation: code that previously took months to write can now be produced in a fraction of that time. However, a developer who manually writes code intrinsically understands what it does and why, and that knowledge is itself a form of quality assurance; delegating the writing to a model forfeits that knowledge, and with it the assurance. That assurance must be re-established through rigorous oversight – precise specification, the domain expertise to recognize that a plausible proposal is unsound and independent verification that the code does what it claims – which demands more effort than traditional manual validation. Ultimately, the fundamental requirement for human domain expertise is unchanged.

The replacement of the legacy CIF parser in *cctbx* served as the primary test case for this methodology. The resulting parser, *xcif*, was validated at archive scale against two independently developed reference parsers (*ucif* and *GEMMI*), while also demonstrating improved speed, reduced memory use and direct integration with *cctbx*’s native data structures. It therefore meets both requirements for a credible software replacement: preserving the established behaviour on which existing software depends and delivering the performance gains that motivated the rewrite.

Completed in about a week of focused expert work, AI-assisted development proves to be a practical and cost-effective tool for renewing foundational scientific software. This approach enables experienced developers to streamline their workflow while remaining reliant on the domain expertise that the technology cannot substitute.

## Supplementary Material

Technical requirements and development plan, design decisions and Supplementary Tables S1 and S2. DOI: 10.1107/S2059798326010211/qe5015sup1.pdf

## Figures and Tables

**Table 1:** Examples where the AI’s first approach to a task was unsuitable or incomplete, the point at which it was identified and how it was resolved

Task	AI’s first approach	Identified at/by	Resolution
Reading values with standard uncertainties	Suggested a fast off-the-shelf routine for turning text into numbers	Design review, adversarial AI critique, confirmed by author	Rejected: that routine cannot handle standard-uncertainty notation such as 1.234 (5). Instead, the value and its parenthesized uncertainty are separated before the number is read.
Deciding which values are numbers	Suggested working out each value’s type – number or text – while reading the file	Design review, adversarial AI critique; author chose a different resolution than the critic proposed	Rejected: this is unreliable in columns that mix numbers with the . and ? markers for missing values. Values are kept exactly as written and turned into numbers only when a program asks for them.
How a loop’s values are stored	Both AI models preferred storing the values column by column, expecting that to make reading a whole column faster	Design review – author, overruling the preference of both AI models	Overruled in favour of storing values row by row – the order they appear in the file – so they can be written down in one pass instead of being sorted into separate columns first
Reserving memory for large loops	Added a rule of thumb that guessed a loop’s size from how much of the file was left to read	Performance testing, benchmark result, interpreted by human	Removed: the guess was inadequate for the Chemical Component Dictionary, which is tens of thousands of small data blocks rather than a few large ones, so it set aside far too much memory
Decimal point under different regional settings	Noted in the design notes that this had to be handled, but left the safeguard out of the code	AI-assisted code review, AI-flagged; human-validated	Corrected: numbers are now always read with ‘.’ as the decimal point, whatever the computer’s regional settings

## Data Availability

*xcif* is available as part of *cctbx* from version 2026.4: https://github.com/cctbx/cctbx_project/releases/tag/v2026.4. The implementation was merged as pull request #1144 to cctbx/cctbx_project. All benchmarking results reported here were obtained with *cctbx* v2026.5; the corresponding source is archived at Software Heritage: https://archive.softwareheritage.org/swh:1:dir:27e35fd1661212639e1ee7c408894ba029812bb5;origin=https://github.com/cctbx/cctbx_project;visit=swh:1:snp:f3969fdb9c837b05718bc6e6bd85ac97a7605dae;anchor=swh:1:rel:ffecbb2eb7f5480b6bb326feea2e7d4eccdb52bf. The planning prompt, development roadmap and design-decisions log are provided as Supplementary Information S1 and S2. The benchmark scripts are available within *cctbx* in cctbx_project/xcif/benchmarks.

## Footnotes

1 https://www.wwpdb.org/news/news?year=2026#69ebcdca1103c56d3f444159.

2 *cctbx*’s core numerical array type, used throughout the library to hold coordinates and other numerical data.

3 The in-memory representation of a parsed CIF that existing *cctbx* tools expect.

4 https://github.com/project-gemmi/mmcif-benchmark.
