# Supplementary material for: Experiences developing structural biology tools with AI coding assistance
Source: Acta Crystallogr D Struct Biol. 2026 Oct 19;82(Pt 11):1251–9. doi: 10.1107/S2059798326010211 (PMC13717824; doi:10.1107/S2059798326010211)
Supplement: Supplementary file 1 [file d-82-01251-sup1.pdf]

STRUCTURAL BIOLOGY

Volume 82 (2026)

Supporting information for article:

Experiences developing structural biology tools with AI coding assistance

Oleg V. Sobolev, Dorothee Liebschner and Paul D. Adams

S1. The technical requirements and development plan

The following is the complete verbatim prompt provided to the large language model (LLM) after step 14 to guide the development of the *xcif* parser..

MISSION

You are an expert C++ performance engineer and structural biology software developer. Your objective is to build `xcif`, the fastest mmCIF parser in the world, as a drop-in replacement for the `ucif` parser in the CCTBX ecosystem. It must outperform GEMMI while maintaining 100% API compatibility with `iotbx.cif.reader`.

HARD CONSTRAINTS (NON-NEGOTIABLE)

1. **C++ Standard:** Strictly C++14. Do NOT use C++17/20 features (no `std::string_view`, no `std::from_chars`, no structured bindings).
2. **Build System:** Must integrate natively into CCTBX using `libtbx` and `SConscript`. Do NOT generate `CMakeLists.txt` or `Makefile`s.
3. **Python Bindings:** Strictly `Boost.Python`. Do NOT use `pybind11`, `nanobind`, or `ctypes`.
4. **Testing Framework:** No external frameworks. C++ tests are standalone executables returning 0 on success. Python tests use standard `assert` and are registered in `libtbx.run_tests_parallel`. Do NOT use `pytest`, `GTest`, or `Catch2`.
5. **Memory & I/O:** The parser must be zero-copy wherever possible, utilizing memory-mapped file reading and custom string interning at the C++/Python boundary to stay within strict RSS memory limits.
6. **Target Platforms:** Linux, macOS, and Windows. The `mmap` abstraction must support POSIX `mmap` (Linux/macOS) and `CreateFileMapping`/`MapViewOfFile` (Windows) from the start. Do NOT defer Windows support.
7. **API Compatibility Scope:** Initial compatibility targets the core read path of `iotbx.cif.reader`: block/loop/save-frame access, `flex.double`/`flex.int`/`flex.std_string` column extraction, and `as_double`/`as_int`/`as_string` value accessors. Full `miller` array and CIF dictionary support is deferred to a future phase.
8. **Benchmark Target:** The primary benchmark is wall-clock parse time and peak RSS on PDB entry `4v9d.cif` (large ribosome structure, ~300 MB). Secondary benchmarks are `1ubs.cif` (small, fast-path validation) and `3j3q.cif` (medium, multi-model). GEMMI's `cif::read_file` is the performance baseline to beat.

BEHAVIORAL RULES

- Do NOT apologize, repeat constraints back, or add preamble. Get straight to work.
- Do NOT output more than ~300 lines of code per response. If a step requires more, break it into sub-steps and confirm each before continuing.
- Include the full file path as a comment on the first line of every code block (e.g., `// cctbx_project/xcif/tokenizer.h`).

- When proposing architectural decisions, present them as a concise table of options with pros/cons. Do not write essays.

OUTPUT REQUIREMENTS

Test Instructions

After every step that produces test files, output a `## How to Test` section with exact shell commands I can copy-paste to build and run the tests myself. Assume a standard CCTBX development environment with `libtbx.SConscript` available. Include both C++ compilation/run commands and Python test commands as applicable.

Session State Block

At the end of **every** response, output a fenced block exactly like this:

STATE:

Completed: Steps [list] Key decisions: [summary of interception point outcomes, if any] Current step: [number] Next action: [what you will do next, or what you are waiting on from me] Known issues: [any open problems, or "None"]

This block is your handoff artifact. If I start a new session, I will paste your most recent STATE block back to you. Use it as your sole source of project context.

RULES OF ENGAGEMENT: MICRO-TDD & INTERCEPTION POINTS

You will execute this project strictly via Test-Driven Development (TDD). You will write the tests for a specific layer, ensure they fail/do not compile, and then write the implementation to make them pass.

You must execute the 15-Step Roadmap sequentially. However, you are **STRICTLY FORBIDDEN** from proceeding past specific steps without stopping for a "Technical Design Review." At these Interception Points, you must propose your architectural approach as a concise options table with pros/cons (especially regarding C++14, cross-platform support, and performance), and wait for my explicit approval.

THE 15-STEP ROADMAP

Phase I: Scaffolding & Tokenizer

- **Step 1:** Module scaffolding (`cctbx_project/xcif/` with `__init__.py`, `SConscript` stub, `run_tests.py`).

- **Step 2:** C++ test infrastructure (`xcif/regression/cpp/test_utils.h` with tracking macros).
- **Step 3:** Write C++ tokenizer tests (`tst_tokenizer.cpp` covering CIF tokens and edge cases). □ **INTERCEPTION POINT 1: Tokenizer Memory Model** (Stop and discuss zero-copy token representations, cross-platform memory-mapped I/O abstraction for Linux/macOS/Windows, and line/column tracking without branching before implementing Step 4).
- **Step 4:** Implement C++ tokenizer (`tokenizer.h`/`.cpp`).

Phase II: Parser Core & Data Model

- **Step 5:** Write C++ parser and data model tests (`tst_data_model.cpp`, `tst_error_handling.cpp`). □ **INTERCEPTION POINT 2: Data Model & Hash Strategy** (Stop and discuss case-insensitive string hashing and tag storage before implementing Step 6).
- **Step 6:** Implement C++ parser core (blocks, loops, save frames, error detection).
- **Step 7:** Write C++ numeric/value interpretation tests (`tst_numeric.cpp` including SU extraction). □ **INTERCEPTION POINT 3: Numeric Parsing Strategy** (Stop and discuss `strtod` alternatives, SIMD fallbacks, and integer SU extraction before implementing Step 8).
- **Step 8:** Implement C++ numeric parsing.

Phase III: The Python Boundary

- **Step 9:** Write Python binding tests (`tst_bindings.py`, `tst_memory.py`, `tst_gil_release.py`, `tst_string_interning.py`). □ **INTERCEPTION POINT 4: Python-C++ Memory Bridge** (Stop and discuss CPython string interning via Boost, GIL release, and direct `flex.double`/`flex.int` population before implementing Step 10).
- **Step 10:** Implement Boost.Python bindings (`xcif_ext.cpp`) and the Builder protocol.

Phase IV: API Compatibility & Integration

- **Step 11:** Write API compatibility and round-trip tests (`tst_api_compat.py`, `tst_roundtrip.py`). □ **INTERCEPTION POINT 5: I/O and System Integration** (Stop and discuss cross-platform memory-mapped I/O edge cases across Linux/macOS/Windows and `file_object` buffer fallbacks before implementing Step 12).
- **Step 12:** Implement `xcif.reader`, write support, and wire up `iotbx.cif` integration.

Phase V: Validation, Hardening, & Benchmarking

- **Step 13:** Implement `fetch_test_data.py` and run Tier 2 real-world validation tests (`tst_pdb_coordinates.py`, `tst_pdb_sf.py`).
- **Step 14:** Write thread-safety tests (`tst_thread_safety.cpp`) and fuzz targets (`fuzz_target.cpp`).

- **Step 15:** Implement benchmark harness (`run_benchmarks.py`) and optimize until GEMMI is defeated on `4v9d.cif` wall-clock and RSS.

Two things to know going forward:

1. To see std::cout output: run the executable directly — libtbx.run_tests_parallel swallows stdout unless the test fails.
2. Never rm build artifacts manually — if SCons state gets corrupted, run libtbx.configure xcif from the build dir to re-register, then libtbx.scons -j 11 to rebuild.

STATE:

Completed: Steps 1-14 + get_loop_or_row compatibility fix Key decisions: IP1: A/A/A; IP2: B/B/A; IP3: A/A/B; IP4: A/A/C(hybrid); IP5: B/A/A Current step: 14 (complete — 21/21 tests passing, thread safety + fuzz targets) Next action: Step 15 — Implement benchmark harness (run_benchmarks.py) and optimize until GEMMI is defeated on 4v9d.cif wall-clock and RSS Known issues: None

INITIALIZATION COMMAND

Confirm the constraints in one sentence and stage we are on.

S2. Design decisions.

The following was produced at the end of the implementation phase by the coding agent instructed to document the architectural decisions taken at the five interception points. It is therefore a retrospective summary written for documentation purposes, not a transcript of the sessions; the agent's original options tables, presented live at each interception point, were not retained. The options recorded as chosen match the preserved final session-state record and are directly verifiable against the merged implementation, which embodies each choice. The developers, who were party to every decision, reviewed this document for accuracy.

xcif Design Decisions — Technical Reference

This document records the architectural decisions made at each Interception Point during xcif development. Each decision includes the chosen option, full reasoning, and rejected alternatives.

Interception Point 1 - Tokenizer Memory Model: A / A / A

Decision 1 (A): Zero-copy ptr+len tokens

Choice: Tokens are 16-byte structs (const char* ptr, size_t len) pointing directly into the source buffer. No string copies during tokenization.
Reasoning:

- The tokenizer produces millions of tokens for large files. Allocating a std::string per token would dominate parse time.
- ptr+len is the natural output of a character-scanning loop.
- Token text is materialized (via as_str()) only when needed, which is almost never on the hot path.

Decision 2 (A): Memory-mapped I/O deferred to reader layer

Choice: The Tokenizer takes a raw (const char*, size_t) buffer. Memory-mapped file handling is the caller's responsibility (via MappedFile, wired up in the reader layer at Step 12).
Reasoning:

- Keeps the tokenizer a pure computation unit with no I/O dependencies, making it trivially testable with string literals.
- MappedFile is already implemented as a separate RAII wrapper with cross-platform support (POSIX mmap / Windows CreateFileMapping).

Decision 3 (A): Simple line counter

Choice: Track line and column via a simple integer counter incremented on newline characters. No branchless tricks.
Reasoning:

Line/column tracking adds one branch per character in the worst case (inside string scanning loops), but the branch predictor handles this well since newlines are rare relative to other characters.
Correctness and debuggability outweigh the ~1% theoretical throughput gain from SIMD newline counting.

Interception Point 2 - Data Model & Hash Strategy: B / B / A

Decision 1 (B): Case-Insensitive Hash+Equal on string_view Keys

Choice: Custom CIHash and CIEqual functors operating on xcif::string_view. Map type: std::unordered_map<xcif::string_view, T, CIHash, CIEqual>.
Reasoning:

- CIF spec requires case-insensitive tag and block name matching. The lookup mechanism is used on every find_value(), find_loop(), and find_block() call.
- xcif::string_view keys avoid the C++14 limitation where std::unordered_map<std::string>::find() forces a std::string construction from const char*. With string_view as the key type, lookup from const char* constructs only a 16-byte view (stack, zero heap allocation).
- Hash: FNV-1a over tolower() of each byte. Simple, fast, and collision-resistant for the typical CIF tag population (~100–500 tags per block).
- Map keys are string_view pointing into stable owned storage (tag names stored in the Block's tag list or a dedup pool). Lifetime is tied to the Document, which owns all backing memory.
- When C++17 arrives, xcif::string_view becomes std::string_view via the preprocessor alias — no code changes to hash functors or map types.

Rejected alternatives:

(A) Lowercase on insert + lowercase on lookup: Allocates a std::string for every lookup query. Though the allocation cost is negligible at ~200 lookups per block, it's unnecessary complexity (two representations of each tag) when functors achieve the same result with one.
(C) Sorted vector + binary search: O(log n) lookup and O(n) insert. No advantage over hash map for CIF's flat tag namespace.

Decision 2 (B): string_view Value Storage with Document-Owned Buffer

Choice: All values (tag values, loop cell values, block names, tag names) are stored as xcif::string_view pointing into the source buffer. The Document owns the buffer lifetime — either a MappedFile (file input) or a std::string copy (string input).
Reasoning:

Parse-time allocation elimination. A 300MB CIF file like 4v9d.cif contains ~5M values. With std::string storage (Option A), parsing requires ~5M string constructions (even with SSO, each is a memcpy). With string_view, parsing records ~5M 16-byte ptr+len pairs —

effectively free. This is the single largest performance difference vs. GEMMI, which stores std::string copies.

RSS reduction. With std::string storage, peak RSS ≈ file size (mmap) + file size (string copies) ≈ 2×. With string_view, peak RSS ≈ file size (mmap) + metadata overhead ≈ 1.05×. For a 300MB file, this is the difference between ~300MB and ~600MB.

Python synergy. The dominant access pattern is Python → column extraction → flex.double/flex.int. With string_view storage, the path is: mmap buffer → strtod() → flex.double. No intermediate C++ std::string is ever constructed. With std::string storage, the path is: mmap buffer → std::string → strtod() → flex.double — one pointless copy per value.

For string values accessed from Python (e.g., atom names), Boost.Python materializes the string_view directly into a Python str object. The path is: mmap buffer → PyUnicode_FromStringAndSize(). Again, no C++ std::string middleman.

Buffer ownership model:

```
Document
├ MappedFile mapped_     (file input — owns mmap'd pages)
├ std::string owned_buf_ (string input — owns copied buffer)
└ blocks_                (all string_views point into one of the above)
```

The parse(const char*) convenience overload copies the input into owned_buf_ so the Document is self-contained. The parse(MappedFile&&) overload takes ownership of the mapped file.

Rejected alternatives:

- (A) std::string copies: Simple ownership but doubles RSS and adds ~5M unnecessary allocations on the hot path. The "correctness first" argument doesn't hold — string_view is not more error-prone when the Document owns the backing buffer via RAII.
- (C) Hybrid views-then-arena: Unnecessary two-phase complexity. The RAII ownership model achieves the same result with simpler code.

Decision 3 (A): Row-Major Flat Loop Layout

Choice: Loop values stored in a single std::vector<xcif::string_view> in row-major order. Access: values_[row * width + col].

Reasoning:

- Natural parse order. CIF loops are written row-by-row. Row-major storage means values are appended in parse order — a single push_back per value with no scatter.
- Single allocation. One vector with geometric growth, vs. column-major's N vectors (one per tag). For a loop with 20 tags and 100K rows, this is 1 allocation vs. 20.
- Column extraction is not the bottleneck here. Column extraction (stride access across rows) is slightly less cache-friendly than column-major's contiguous access. However, the performance-critical column extraction happens in the Builder layer (Step 10), where the Builder writes directly into flex arrays during parsing — bypassing the Loop data

structure entirely. The Loop::column() method is a convenience API for interactive/non-performance-critical use.
- Simpler invariant. values_.size() % width_ == 0 is a single check that validates the entire loop. Column-major requires checking that all column vectors have equal length.

Rejected alternative:

- (B) Column-major: Would make Loop::column() a direct vector return (O(1) vs. O(n) stride copy), but adds parse-time complexity and more allocations. The Builder protocol in Step 10 makes this optimization moot for the hot path.

Supplemental: xcif::string_view Design

Choice: A minimal C++14 string_view implementation in xcif/include/xcif/string_view.h, conditionally aliased to std::string_view when compiled under C++17 or later.
Reasoning:

- C++14 has no std::string_view. The project needs a non-owning string reference as its vocabulary type for zero-copy token storage, map keys, and value access.
- The implementation provides only the subset of the std::string_view API that xcif actually uses: constructors (ptr+len, const char*, std::string), data()/size()/empty(), operator==/!= (vs string_view, const char*, std::string), operator<, substr(), find(char), begin()/end(), stream output, and explicit conversion to std::string.
- A preprocessor guard (#if __cplusplus >= 201703L) switches the entire type to a using-alias for std::string_view. Migration to C++17 requires zero code changes at call sites — the alias is the only thing that changes.
- Boost.string_view was rejected because its API does not exactly match std::string_view, making the migration less clean. A third-party single-header library was rejected to avoid adding an external dependency.

Buffer ownership and move safety:

- Document stores its backing buffer as std::vector<char> (not std::string) to avoid Small String Optimization (SSO). SSO stores short strings inline in the object; when the object is moved, the inline buffer address changes, invalidating any string_views that pointed into it. std::vector<char> always uses heap storage, so move transfers the heap pointer and all existing string_views remain valid.

Interception Point 3 - Numeric Parsing Strategy: A / A / B

Decision 1 (A): strtod() with stack buffer for double parsing

Choice: Copy the string_view content into a char[32] stack buffer, NUL-terminate, and call strtod(). No heap allocation.
Reasoning:

CIF numeric values are short — typically under 20 characters (e.g. "1.542", "-23.45(12)", "1.5e-3"). A 32-byte stack buffer accommodates all realistic values with room to spare.
strtod() is the C standard library's battle-tested floating-point parser. It handles all IEEE 754 edge cases (subnormals, infinities, rounding) correctly. A hand-rolled parser risks subtle precision bugs that are difficult to diagnose in crystallographic calculations.

The stack copy adds ~2ns per value. strtod() itself takes ~15-30ns. The copy overhead is <10% of the total conversion cost — negligible.
The string_view is not NUL-terminated (it points into a larger buffer), so strtod() cannot be called directly on it. The stack buffer solves this with zero heap allocation.
Locale: CIF always uses '.' as the decimal separator. The implementation ensures the C locale is active (or uses strtod_l where available) to avoid locale-dependent parsing.

Rejected alternatives:

- (B) In-place NUL poke (overwrite the byte after the value with '\0', call strtod, restore): Works for owned_buf_ (writable vector<char>) but fails for MappedFile (read-only pages). Requires two code paths and risks SIGSEGV on mmap'd buffers.
- (C) Custom hand-rolled fast_strtod: Avoids NUL-termination but risks precision bugs. The performance gain (eliminating a memcpy of <20 bytes) is not worth the correctness risk for a library used in crystallographic structure determination.

Decision 2 (A): Backward scan for SU extraction

Choice: On the rare path where a value contains standard uncertainty (e.g. "1.542(3)"), scan backward from the end to find '(', extract the SU digits, truncate the value, then parse both parts.
Reasoning:

- SU values are rare in practice. The vast majority of numeric CIF values (~99%+) are plain numbers without parenthesized uncertainty.
- The common path does a single memchr() for '(' in the value string. If not found (common case), execution falls straight to strtod() with zero additional overhead.
- When SU IS present, it is always at the end of the value string in the format "value(digits)". A backward scan from the closing ')' to the opening '(' extracts the SU digits in one pass.
- The SU scale is determined by counting decimal places in the value portion: su = su_digits * 10^(-decimal_places). For example, "1.542(3)" has 3 decimal places, so su = 3 * 10^-3 = 0.003.

Rejected alternative:

- (B) Single forward pass with inline SU detection: Marginal speedup on the rare SU path, but adds branching complexity to every value parse. Since SU is rare, optimizing the common (no-SU) path is more important.

Decision 3 (B): Hand-rolled integer parser

Choice: A simple hand-written loop for parsing CIF integers: optional sign followed by decimal digits.
Reasoning:

- CIF integer format is trivially simple: [+-]?[0-9]+. A hand-written parser is ~10 lines of code with no edge cases.
- Avoids the NUL-termination overhead that strtol() would require (same stack-buffer dance as strtod).
- For the simple integer format, a hand-written loop is both faster and simpler than strtol() + error checking.
- Throws std::invalid_argument on non-integer input (the caller is expected to know which CIF tags are integers).

Rejected alternative:

- (A) strtol() with stack buffer: Correct but overkill for such a simple format. Adds the same NUL-termination overhead as Decision 1 with no corresponding benefit, since the integer format has none of strtod's complexity (no exponents, no decimal points, no subnormals).

Interception Point 4 - Python-C++ Memory Bridge: A / A / C(hybrid)

Decision 4A (A): shared_ptr<Document> with Aliasing Constructor

Choice: The Document returned by parse() is wrapped in a std::shared_ptr<Document> at the Python boundary. When Python requests a child object (Block, Loop), the binding returns an aliased shared_ptr that points to the child but shares the Document's reference count.
Implementation pattern:

```
// Root document
std::shared_ptr<Document> doc_ptr = std::make_shared<Document>(
    std::move(parse(data, len, source)));

// Block wrapper — aliased to Document's refcount
const Block* raw = &(*doc_ptr)[i];
std::shared_ptr<const Block> blk_ptr(doc_ptr, raw);

// Loop wrapper — also aliased to Document's refcount
const Loop* raw_loop = blk_ptr->find_loop("_atom_site");
std::shared_ptr<const Loop> loop_ptr(doc_ptr, raw_loop);
```

Reasoning:

- Bulletproof safety. The Python user can extract a Loop, delete the Document, delete the Block, and the C++ Document memory stays alive because the Loop's aliased shared_ptr keeps the root reference count > 0.
- Zero hot-path overhead. The C++ parser (Step 6) builds standard std::vector<Block> and std::vector<Loop> with no shared_ptr involvement. The shared_ptr wrapping happens only at the Python boundary when the user requests an object.
- Zero Python complexity. Boost.Python natively understands std::shared_ptr. No custodian_and_ward lifecycle policies needed.
- Every wrapper at every depth increments the same atomic refcount (the Document's). No chains of shared_ptrs, no nested ref tracking.
- The parse() function returns Document by value. At the binding layer, std::move(result) into make_shared<Document>() to create the root shared_ptr. The parser itself is untouched.

Rejected alternatives:

- (B) return_internal_reference<> (Boost.Python custodian/ward): Zero overhead but only works for direct parent→child references. Nested chains (doc→block→loop) require manual ward chaining, which is fragile and error-prone.
- (C) Copy all strings to Python at the boundary: No lifetime issues but defeats zero-copy for string access. More memory for large files. Still needs a solution for Loop/Block wrapper lifetime.

Decision 4B (A): GIL Release for parse() and parse_file() Only

Choice: Release the GIL during the C++ parsing work in parse() and parse_file() using a standard RAII scoped release (Py_BEGIN_ALLOW_THREADS / Py_END_ALLOW_THREADS). Do NOT release the GIL during flex column extraction in the initial implementation.
Reasoning:

Correct bottleneck targeting. Parsing a 300 MB CIF file is seconds of CPU work. Column extraction (iterating a std::vector and creating Python objects) is microseconds by comparison. Releasing the GIL there would add complexity for negligible gain.
flex allocation requires the GIL. flex.double ultimately calls PyMem_Malloc through Boost.Python's object model. Releasing the GIL during column extraction would require splitting a simple loop into two phases (pre-allocate raw buffer without GIL, wrap into flex with GIL) — unnecessary complexity.
Simple implementation: one Py_BEGIN_ALLOW_THREADS / Py_END_ALLOW_THREADS pair per entry point.
GIL contention during column extraction will be profiled during Phase V benchmarking. If data proves it is a bottleneck, GIL release will be extended then.

Rejected alternative:

- (B) Release GIL for parse + all flex column extraction: Full pipeline parallelism but requires verifying that flex array allocation is GIL-safe (it isn't — it allocates Python-managed memory). Premature optimization without profiling data.

Decision 4C (C, hybrid): Schema-Aware Tag Heuristic with CPython Native Interning

Choice: A two-tier interning strategy:
1. AST keys (tag names, block names): ALWAYS intern using PyUnicode_InternInPlace when returning to Python.
2. Loop values: Intern ONLY when the parent tag is a known categorical mmCIF tag. All other values are returned as standard un-interned Python strings.

Known categorical tags (compile-time set):
- _atom_site.label_comp_id (residue name: ALA, GLY, ...)
- _atom_site.type_symbol (element: C, N, O, S, ...)
- _atom_site.label_atom_id (atom name: CA, CB, N, ...)
- _atom_site.label_asym_id (chain ID: A, B, ...)
- _atom_site.label_entity_id (entity ID: 1, 2, ...)
- _atom_site.label_alt_id (alt conformation: ., A, B)
- _atom_site.auth_comp_id (author residue name)

- _atom_site.auth_atom_id (author atom name)
- _atom_site.auth_asym_id (author chain ID)

Implementation: A C++ std::unordered_set<xcif::string_view, CIHash, CIEqual> containing the known tags above. When returning a loop column value to Python, check the column's tag against this set. If it matches, call PyUnicode_InternInPlace on the resulting Python string. If not, return a standard PyUnicode.

Reasoning:

- Avoids poisoning the CPython intern table. Coordinate values (27.340, 24.430, 2.614) are unique per-atom — interning them wastes cycles on hash lookups that never hit and bloats CPython's global intern dict.
- Surgically targets low-cardinality columns. A typical mmCIF file has ~20 unique element symbols, ~20 residue names, ~10 chain IDs. These strings repeat millions of times across atoms. Interning them saves significant memory (one Python str object per unique value, not one per atom).
- Tag names are always interned. There are typically ~20-50 unique tags in a CIF file, accessed repeatedly as Python dict keys. Interning is cheap (few tags) and high-value (O(1) pointer equality in Python dicts).
- The known-tag set is small (~10 entries). For sets this small, a flat const char* array with linear scan may be faster than hashing (cache-friendly, no allocator overhead). Start with the flat array; switch to unordered_set only if the list grows past ~30 entries.
- Non-mmCIF files (small molecule CIF, CIF dictionaries, custom schemas) get correct behavior — values simply aren't interned. This is the safe default. No performance penalty, just slightly higher memory for unusual categorical columns.

Rejected alternatives:

- (A) PyUnicode_InternInPlace on every string: Wastes cycles interning unique numeric values that never repeat. For a 300 MB file with ~5M values, the intern hash table lookup overhead is measurable.
- (B) Intern tag names only, values never: Misses the biggest memory savings opportunity (element symbols and residue names repeat millions of times in large structures).

Interception Point 5 - I/O and System Integration: B / A / A

Decision 1 (B): Existing MappedFile wrapper + madvise(MADV_SEQUENTIAL)

Choice: The existing cross-platform MappedFile RAII wrapper handles all baseline edge cases (empty files return empty buffer without mmap; >2GB files use 64-bit size_t). Additionally, after mapping, issue a sequential access hint to the kernel:

- Linux/macOS: posix_madvise(addr, len, POSIX_MADV_SEQUENTIAL)
- Windows: FILE_FLAG_SEQUENTIAL_SCAN passed to CreateFile

Reasoning:

- The tokenizer reads strictly forward from byte 0 to EOF. The SEQUENTIAL hint tells the kernel to aggressively pre-fetch pages ahead of the read position and drop already-read pages from the page cache.
- On large files (300MB+ CIF entries like 4v9d.cif), this prevents page cache thrashing and reduces peak RSS by allowing the kernel to reclaim pages behind the read cursor.

- Cost: ~3 lines per platform in mapped_file.h. Zero runtime overhead — the hint is a one-time syscall after mmap.
- macOS note: use posix_madvise(), not madvise() — the latter exists but has different semantics on Darwin.

Rejected alternatives:

- (A) Baseline only (no madvise): Correct but leaves free performance on the table. The kernel defaults to a generic access pattern assumption, which under-fetches for sequential reads and over-retains pages for random-looking access.
- (C) Threshold-based mmap (mmap only files >64KB): Premature micro-optimization. The kernel overhead of mapping a 4KB file is negligible compared to the Python interpreter overhead already being paid. Adds a second code path for no measurable gain.

Decision 2 (A): file_object fallback via .read() in Python

Choice: xcif.reader accepts file_path, input_string, or file_object. For file_object: call .read() in Python, pass the resulting string to C++ parse() (which copies into owned_buf_). No new C++ code required.

Reasoning:

The tokenizer's contract is (const char*, size_t) — a contiguous buffer (IP1 Decision 2). Chunked reading would break this contract and require the tokenizer to handle state across buffer boundaries.

The buffer-boundary problem is severe: a semicolon-delimited text field or a long quoted string could be sliced mid-token by a chunk boundary. Handling this requires a complex state machine that would slow the tight inner loop for ALL files, not just file_object inputs.

file_object usage is rare compared to file_path (which uses mmap). The .read() path already exists: parse(const char*) copies into owned_buf_ (std::vector<char>), giving the Document full ownership.

Typical file_object use cases (gzip.open, StringIO) involve files that easily fit in memory — the extra copy is irrelevant.

Rejected alternative:

- (B) Chunked streaming reader via Boost.Python: Avoids full materialization in Python but forces the tokenizer to handle cross-boundary token splits. The complexity is severe (every token type — quoted strings, semicolon fields, comments — needs boundary-aware logic), and the benefit is marginal since files passed via file_object are almost always small enough to .read() in one shot.

Decision 3 (A): Thin Python wrapper with lazy adapter

Choice: A thin Python class in xcif/__init__.py that mirrors the iotbx.cif.reader constructor signature (file_path, file_object, input_string, raise_if_errors, strict). Delegates to xcif_ext.parse / parse_file. Returns a model via a lazy adapter layer that wraps xcif Document/Block/Loop to match the iotbx.cif.model dict-like API (model["block"]["_tag"]).

Implementation: The adapter is a pure Python layer of MutableMapping subclasses (_cif_adapter, _block_adapter, _loop_adapter) that translate dict-style access into xcif_ext method calls. No C++ changes required. Python objects are materialized only when accessed (lazy), not during parsing.

Reasoning:

API compatibility is non-negotiable. The iotbx.cif.model interface (dict-like block/tag/loop access, flex.std_string column returns, case-insensitive lookups) is used throughout the CCTBX ecosystem. Breaking it would prevent adoption regardless of performance.

The adapter adds one Python method call of overhead per access, which is invisible compared to the work callers do with the data (building crystal structures, computing maps, etc.).

Preserves GIL release during parse (IP4B): the C++ parser runs without the GIL, builds the complete AST, then the adapter translates on demand in Python.

Preserves zero-copy C++ AST (IP2B): string_view values in the C++ Document are only materialized to Python str objects when the adapter's __getitem__ is called.

Rejected alternatives:

(B) Expose xcif's native API directly (no adapter): Faster integration but breaks API compatibility. Users would need doc[0].find_value("_tag") instead of model["block"]["_tag"]. This defeats the "drop-in replacement" goal.

Python builder callbacks during parse: Initially considered as an implementation variant of Option A. Rejected because it conflicts with IP4B (GIL must be held for Python callbacks, preventing GIL release during parse) and IP2B (Python str objects must be created per-value during parse, defeating zero-copy). On a 300MB file, ~10M Python function callbacks would make xcif slower than the existing iotbx.cif C parser which already uses this pattern.

Table S1 Parse time. Columns: ucif and `xcif_compat` both produce the `iotbx.cif.model`; Gemmi and `xcif_native` both produce a native document.

File	Size (MB)	ucif	xcif_compat	Gemmi	xcif_native
4fxz.cif	0.4	53.1 ms	34.4 ms	1.7 ms	1.3 ms
1cjb.cif	0.8	75.1 ms	38.0 ms	2.9 ms	2.3 ms
3j3q.cif	230.6	16.66 s	1.68 s	928.4 ms	610.4 ms
r4v9psf.ent	248.3	10.62 s	1.25 s	729.1 ms	520.3 ms
r5rhysf.ent	1066.1	210.43 s	5.07 s	3.58 s	2.58 s
r5robsf.ent	3293.6	—	20.13 s	12.44 s	8.13 s
components.cif	479.5	201.10 s	173.89 s	2.80 s	1.79 s

Table S2 Peak resident set size (MB).

File	Size (MB)	ucif	xcif_compat	Gemmi	xcif_native
4fxz.cif	0.4	162	74	20	47
1cjb.cif	0.8	224	98	25	57
3j3q.cif	230.6	14840	3960	2827	2333
r4v9psf.ent	248.3	14122	2872	2567	1980
r5rhysf.ent	1066.1	18404	10285	7079	5201
r5robsf.ent	3293.6	—	20217	15225	14413
components.cif	479.5	14911	6758	3568	3025
